# AI and Computational Methods for Modelling, Simulations and Optimizing of Advanced Systems: Innovations in Complexity

**DOI:** 10.3390/ma18174112

**Published:** 2025-09-01

**Authors:** Marcin Sosnowski, Jaroslaw Krzywanski, Karolina Grabowska, Dorian Skrobek, Ghulam Moeen Uddin, Cezary Kozlowski, Jacek Przybylski, Malgorzata Deska

**Affiliations:** 1 Faculty of Science and Technology, Jan Dlugosz University in Czestochowa, Armii Krajowej 13/15, 42-200 Czestochowa, Poland; j.krzywanski@ujd.edu.pl (J.K.); k.grabowska@ujd.edu.pl (K.G.); d.skrobek@ujd.edu.pl (D.S.); c.kozlowski@ujd.edu.pl (C.K.); j.przybylski@ujd.edu.pl (J.P.); m.deska@ujd.edu.pl (M.D.); 2 Department of Mechanical Engineering, University of Engineering and Technology, Lahore 54890, Punjab, Pakistan; ghulammoeenuddin@uet.edu.pk

The rapid evolution of artificial intelligence (AI) and computational methods has transformed the landscape of modeling, simulation, and optimization, particularly for complex, interdisciplinary systems. As scientific challenges grow increasingly multifaceted—from energy systems to advanced engineered materials, and from multi-scale biological phenomena to data-driven industrial solutions—the integration of AI with traditional and novel computational approaches continues to produce breakthrough advances.

The presented MDPI Topic “AI and Computational Methods for Modelling, Simulations and Optimizing of Advanced Systems: Innovations in Complexity” brings together 40 original research articles that span foundational algorithmic innovations to impactful case studies in science and engineering. This Topic responds to the growing demand for robust, adaptable, and interpretable computational methods capable of addressing real-world, high-dimensional, and nonlinear problems. Here, we present a brief overview of recent advances, persistent knowledge gaps, the contributions of this Topic, and future research directions.

Recent years have seen remarkable synergies between physics-based modeling, traditional numerical methods (FEM, FDM, etc.), and data-driven or hybrid AI approaches. Physics-guided and domain-informed machine learning has substantially improved the interpretability and accuracy of models for complex, chaotic, or data-scarce systems. AI-enhanced solvers and hybrid frameworks demonstrably outperform classical techniques in predicting nonlinear dynamics, optimizing system performance, and discovering new physical laws [1,2,3]. Generative AI and reinforcement learning are beginning to revolutionize optimization in engineering design, energy management, and manufacturing through the capacity for on-the-fly adaptation and real-time learning [4]. Surrogate modeling, hyper-heuristics, and neural-based metaheuristics (evolutionary algorithms hybridized with ML) now enable tractable optimization of previously intractable, high-dimensional design spaces [5,6].

The field of complexity science has profoundly benefited from data-driven methods that can extract hidden patterns and guide systemic interventions across scales. Advances in knowledge graph construction, multi-scale simulation, and agent-based modeling directly address pressing challenges in fields such as healthcare, logistics, and environmental management [7,8].

Despite remarkable progress, the community faces several inherent challenges:

- Interpretability and Transparency: As models grow in sophistication and autonomy, ensuring their interpretability, especially in critical domains (energy, health, finance), is necessary for trust and regulatory acceptance [6].
- Robustness and Scalability: Many AI-enhanced optimization tools excel on test cases but struggle with generalization, transferability, and scaling to industrially relevant, real-world problems [8,9].
- Integration with Domain Knowledge: It remains challenging to systematically incorporate physical laws, constraints, and expert insights directly into learning architectures without loss of flexibility [2,7,9].
- Benchmarking and Reproducibility: A proliferation of methods and benchmarks complicates fair comparisons, reproducibility, and consistent progress assessment across domains [3,10,11].

This Topic, “AI and Computational Methods for Modelling, Simulations and Optimizing of Advanced Systems: Innovations in Complexity”, addresses multiple facets of these knowledge gaps through research that proposes and validates novel hybrid algorithms uniting classical optimization (GA, PSO, ACO) with neural, evolutionary, or reinforcement learning components; presents case studies of real-world applications (e.g., material design, smart grid optimization, energy storage, process control) where advanced AI-based simulation and modeling drive measurable improvements; demonstrates approaches for explainability and physical interpretability in deep learning for dynamical and multi-scale systems; advances the theoretical underpinnings of network science, multifractal analysis, and information-theoretic methods with computational innovation; and benchmarks and evaluates emerging methods with careful attention to open-source data availability and reproducible research practices. The published articles are distributed among the journals as indicated in Table 1.

The contributions to the Topic “AI and Computational Methods for Modelling, Simulations and Optimizing of Advanced Systems: Innovations in Complexity” are listed below:

## Figures and Tables

**Table 1:** The distribution of articles among the journals.

Journal	No. of Papers
*Energies*	11
*Entropy*	9
*Materials*	9
*Fractal and Fractional*	4
*Machine Learning & Knowledge Extraction (MAKE)*	4
*Algorithms*	3
